# Distortion dependent intersystem crossing: A femtosecond time-resolved photoelectron spectroscopy study of benzene, toluene, and p-xylene

**DOI:** 10.1063/1.4977735

**Published:** 2017-02-28

**Authors:** Anne B. Stephansen, Theis I. Sølling

**Affiliations:** Department of Chemistry, University of Copenhagen, Universitetsparken 5, DK-2100 København Ø, Denmark

## Abstract

The competition between ultrafast intersystem crossing and internal conversion in benzene, toluene, and p-xylene is investigated with time-resolved photoelectron spectroscopy and quantum chemical calculations. By exciting to S_2_ out-of-plane symmetry breaking, distortions are activated at early times whereupon spin-forbidden intersystem crossing becomes (partly) allowed. Natural bond orbital analysis suggests that the pinnacle carbon atoms distorting from the aromatic plane change hybridization between the planar Franck-Condon geometry and the deformed (boat-shaped) S_2_ equilibrium geometry. The effect is observed to increase in the presence of methyl-groups on the pinnacle carbon-atoms, where largest extents of σ and π orbital-mixing are observed. This is fully consistent with the time-resolved spectroscopy data: Toluene and *p-*xylene show evidence for ultrafast triplet formation competing with internal conversion, while benzene appears to only decay via internal conversion within the singlet manifold. For toluene and *p*-xylene, internal conversion to S_1_ and intersystem crossing to T_3_ occur within the time-resolution of our instrument. The receiver triplet state (T_3_) is found to undergo internal conversion in the triplet manifold within ≈100–150 fs (toluene) or ≈180–200 fs (p-xylene) as demonstrated by matching rise and decay components of upper and lower triplet states. Overall, the effect of methylation is found to both increase the intersystem crossing probability and direct the molecular axis of the excited state dynamics.

## I. INTRODUCTION

**FIG. 1.**
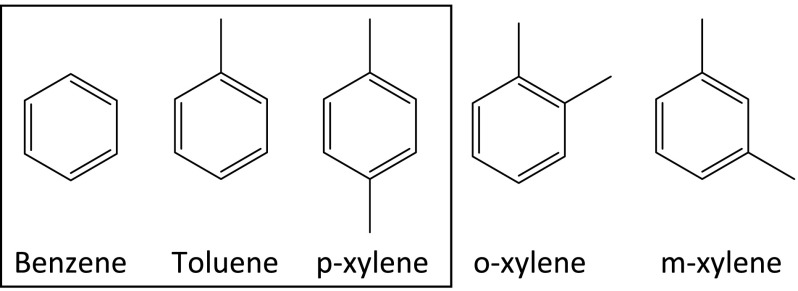
Structures of benzene, toluene, and the xylene isomers.

Benzene and its singly and doubly methylated derivatives toluene and xylenes (Figure 1) exhibit similar photoinduced properties, but the presence of methyl groups entails subtle differences between the three substances that mark themselves in ultrafast time-resolved investigations. Photoinduced properties of benzene have been extensively studied for decades, yet controversy on certain aspects persists.^1–7^ One example is the observation of ultrafast intersystem crossing (ISC) competing with internal conversion (IC).^1,3,4^ ISC, the non-radiative spin-forbidden transition between two electronic states of different spin-multiplicity, is conventionally expected to be slow compared to IC, the equivalent transition between two states of the same multiplicity. Electron spin-flip requires that a magnetic torque, such as spin-orbit coupling, acts on the spin:^8^ Spin-orbit couplings are frequently evaluated with the Breit-Pauli Hamiltonian to which the associated expectation value scales with the fourth power of the nuclear charge,^9^ demonstrating the commonly anticipated sensitivity of spin-orbit couplings and ISC efficiencies towards the presence of heavy atoms. In organic chemistry where heavy atoms are sparse, the probability of an ISC process is often estimated from El-Sayed's rule^10^ stating that ISC becomes partly spin-allowed if it is paired with a simultaneous change in orbital angular momentum thereby ensuring overall conservation of angular momentum. This propensity rule rationalizes why ISC processes between for instance ^3^(n,π*) and ^1^(π, π*) states can be efficient in some hetero-atom containing organic molecules.^10^ Benzene exhibits neither heavy atoms to enhance spin-orbit couplings nor heteroatoms to facilitate classic El-Sayed type transitions. The only immediately apparent ISC transition ensuring angular momentum conservation involves σ-orbitals; however, σ and π orbitals do not mix in planar geometries like that of ground state benzene.^11^ Ultrafast ISC in benzene is therefore not immediately expected. In this contribution, we assess the competition between IC and ISC as S_2_ deactivation pathways for benzene, toluene, and *p-*xylene (boxed in Figure 1) via femtosecond (fs) time-resolved photoelectron spectroscopy (TRPES) investigations. The inclusion of methylated analogues reveals otherwise elusive information on the unexpected IC vs. ISC competition.

The conventional notion that ISC fundamentally is slower than IC has been challenged by several experimental observations presented during the past decade, where ISC processes have been reported to occur on the timescale of molecular vibrations (fs to picoseconds^12^). The (non-exhaustive) list of organic systems reported to exhibit ultrafast ISC includes a variety of molecules such as nitroaromatic compounds,^13–24^ some natural nucleobases^25–28^ but particularly the thionated analogues,^26,29–35^ molecules known from photovoltaic applications such as perylene diimide,^36^ poly-methines,^37^ perylene bismide,^38^ and familiar organic solvents such as benzene,^3,4^ *ortho-* and *meta-*xylenes,^39–42^ and small liquid esters;^43^ thus, molecules vary in both size and functionality. This diversity suggests that ultrafast ISC could be a general phenomenon. However, in comparison to IC, the importance and behavior of ISC in the ultrafast time regime are much less established. For IC, it has been shown that the ultrafast nature of the process rests on large degrees of non-ergodicity;^44,45^ in other words, only a few vibrational modes are active during the transition. If the vibrations that are activated upon photon absorption couple different electronic surfaces, the possibility of an ultrafast transition is available and can occur within the timescale of the respective vibrational period. IC and ISC are fundamentally similar,^9^ and thus, ISC should also be able to occur within a vibrational period. Yet, the requirement of angular momentum conservation persists, and an ultrafast ISC process should preferentially occur along vibrational coordinates that ensure spin-flip compensation.

**FIG. 2.**
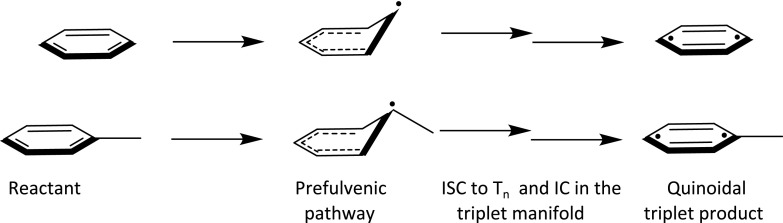
Illustration of a partly allowed intersystem crossing process in planar aromatic hydrocarbons. Out-of-plane distortions are required for the spin-forbidden process to become partly allowed; the pre-fulvene coordinate is a possible mechanism and posited to play a large role for benzene and its derivatives. Both the frequency of the prefulvene coordinate and the final quinoidal triplet product depend on the presence of methyl groups on the pinnacle carbon.

Planar aromatic hydrocarbons like benzene must therefore activate symmetry breaking modes^6,11^ that allow mixing of σ and π orbitals at early times for ultrafast ISC to occur. This entails initial activation of out-of-plane modes forming pseudo-radicaloid species. Figure 2 shows an example of such distortion, where the prefulvene-like coordinate is exemplified as the out-of-plane deformation mode. Such mechanism exactly corresponds to the ISC mechanism suggested by the groups of Fielding and Worth for the S_1_ → T_2_ ISC in benzene.^1,3,4^ The frequencies of such modes will depend on the presence of methyl-substituents on the pinnacle carbon atom(s) distorting from the molecular plane.^46,47^ The pinnacle carbon atoms are expected to be the ones carrying the methyl-substituents due to the stabilizing effect of methyl-groups on the un-paired electron of the quasi-radicaloid prefulvene-like structure.^48^ This reasoning is fully consistent with the observation that the lowest triplet states of toluene and p-xylene exhibit quinoidal structures (Figure 2) with the methyl-groups positioned to support the diradicaloid species as found by EPR measurements^49,50^ and quantum chemical calculations.^48^ Quinoidal triplet structures of benzene derivatives have further been observed by time-resolved electron diffraction experiments by Zewail and coworkers.^51^ Triplet energies and the frequency of the modes that could mediate ISC in benzene and its derivatives are therefore expected to be sensitive towards methylation.

The rich photophysics of benzene can briefly be summarized as follows: Resonant excitation to S_1_ is followed by fluorescence and a slow (nanosecond timescale) ISC pathway,^52–56^ and these channels are often referred to as channel 1 and channel 2, respectively. When the excitation energy is increased 3000 cm^−1^ above the S_1_ onset, the fluorescence yield decreases drastically due to activation of a third channel (the controversial “channel 3” first reported by Callomon in the 1960s^57^), which has been identified as an ultrafast non-radiative process of IC and/or ISC character.^58–62^ The crossing points to both the triplet manifold and the singlet ground state are reached via prefulvenic out-of-plane distortion positioned just behind an activation barrier of ≈3000 cm^−1^.^1,3,4,6^ With 12 permutational isomers of the minimum energy prefulvenic conical intersection that can be inter-connected via various conformational routes, prefulvene-like conical intersections are posited to play a significant role for the photoinduced processes of benzene.^6,63,64^ A prefulvene-like conical intersection is also proposed to mediate IC from S_2_ to S_1_ in both benzene and toluene, which is found to occur on a 40–60 fs timescale.^47,65–67^ The involvement of a prefulvene-like conical intersection for the S_2_/S_1_ IC is in line with the theoretical finding that the equilibrium structure of S_2_ is boat-shaped^64^ along with the notion that a boat-shaped geometry constitutes the crossing point between two prefulvenic isomers.^6^ Importantly, the boat-shaped equilibrium structure of S_2_ (Ref. 64) differs from the planar Franck-Condon geometry; this indicates that initial relaxation upon excitation to S_2_ primarily should activate boat-type modes.

Newer time-resolved mass spectrometry (TRMS) studies on toluene and *o*, *m*, and *p*-xylene along with^46,47^ TRPES studies on *o-* and *m*-xylene explored non-radiative decays from S_3_.^40–42^ These studies found that the S_3_→S_2_ transition proceeds via a full-boat distortion, while S_2_ → S_1_ proceeds via half-boat distortions.^46,47^ These interpretations were based on correlations between the observed decay rates and the position of methyl groups. Full-boat distortions are slowed down by methyl-groups in *p*-position, which accordingly slows down the IC process for *p*-substituted systems compared to toluene and the *o*-/*m*-substituted analogues. The frequency of half-boat (prefulvene-like) distortions on the other hand is more affected by *o*-substitution than *p-/m*-substitution due to steric congestion, which is in agreement with the observation of slower S_2_ → S_1_ rates for *o*-xylene as compared to *p*-xylene and toluene. The TRPES studies on *o-*xylene also invoked ISC from S_2_ to T_3_ as a possible competing deactivation channel to explain an observed slightly slower decay component.^40–42^ Qiu *et al*. also report observation of S_2_ → T_3_ ISC in benzene^67^ while other studies assessing the S_2_ deactivation of benzene do not report observations on the ISC pathway.^65,66^

In this contribution, we explore the S_2_ → T_3_ channel further and assess what effect one or two methyl groups have on the dynamics of the benzene-skeleton. Upon excitation to S_2_ early activation of out-of-plane distortions is expected. IC vs ISC competitions and the role of methylation are considered through comparison of the S_2_ decay for benzene, toluene, and *p*-xylene measured by fs TRPES. The investigation is supported by quantum chemical calculations primarily assessing (changes in) hybridization of the bonding carbon-orbitals on the S_2_ surface. The key motivation is to increase the understanding of ultrafast ISC and what effects promote ISC in a non-El-Sayed system.

## II. EXPERIMENTAL

The setup for fs TRPES experiments consists of a fs pulsed laser system and a photoelectron spectrometer employing velocity map imaging^68^ (VMI) detection and has been described in detail previously.^69,70^ Briefly, the laser system consists of a Ti:Sapphire oscillator (Tsunami, Spectra Physics) and a regenerative amplifier system (Spitfire, Spectra-Physics) that eventually outputs 798 nm pulses of approximately 140 fs duration with an intensity of ca. 1 W at 1 kHz repetition rate. The pulses were split into two: 50% were used to generate the fourth harmonic of the fundamental (6.2 eV = 200 nm, ca. 1 mW intensity), which was used as pump pulse. The remaining 50% were sent through an optical parametric amplifier (TOPAS-C, Light Conversion), which was set to output pulses at three different energies 4.44 eV (279 nm, intensity of ca. 1.7 mW), 4.35 eV (285 nm, 1.9 mW), and 4.2 eV (295 nm, intensity of ca. 2.5 mW) used as the probe pulses in the experiments on benzene, toluene, and *p*-xylene, respectively. The two beams were collected and focused collinearly into the region of interaction with the molecular beam. The cross-correlation between the two pulses was approximately 150 fs.

The molecular beam of either benzene, toluene, or *p-*xylene was generated by bubbling helium at approximately 2 bars over a cartridge containing the sample molecules, and the resulting gaseous solution was expanded into vacuum through a 1 kHz pulsed Even-Lavie valve. The valve temperature was controlled by a cobber cooling loop containing a mixture of water and glycol to maintain a temperature of 28–30 °C. The expansion was subsequently collimated by a skimmer focusing the molecular beam towards the interaction chamber, where it was intersected at right angles with the laser beams. Upon interaction with the pump pulses (6.2 eV), the molecules were excited slightly above the S_2_ threshold (the resonant S_2_ values are reported to be benzene: 6.03 eV, toluene: 5.83 eV, and p-xylene: 5.68 eV (Refs. 71 and 72)). The S_3_ onsets are >6.7 eV, i.e., well above the pump photon energy.^71^ After a well-defined time-delay, the probe pulses ionized to (mainly) D_0_ positioned 9.24 eV, 8.8 eV, and 8.4 eV above the ground state equilibrium.^71,72^ The generated photoelectrons are focused by classic Eppink-Parker VMI ion-optics.^68^ The photoelectrons were detected by a set of 2D position sensitive MCPs on top of a phosphor screen (Photonis) imaged by a monochrome CCD camera. For each data collection acquisition, equivalent pump-only/probe only data were collected and subsequently subtracted to eliminate scattering noise obscuring the data. The photoelectron images were reconstructed using an inverse Abel transformation and calibrated from signals on dimethyl isopropyl amine and butadiene.^69^

The experiments were corroborated by quantum mechanical calculations. State averaged complete active space self-consistent field (SA-CASSCF) with a 6–31G* basis set and an active space of 6 electrons in 6 orbitals for benzene (denoted [6,6]) and [8,8] for toluene and *p-*xylene was used to optimize equilibrium structures of S_0_, and frequency calculations on the optimized geometries were used to verify that the geometries represent minimum energy structures. The minimum energy S_2_ structure of benzene was optimized on the same level of theory. The S_2_ geometry optimization of toluene and *p*-xylene unfortunately did not converge to minimum energy structures, but either converged to planar structures with two imaginary frequencies (as also previously reported for benzene^64^) or cycled around highly distorted structures in a manner indicating that (cumbersome) full configuration interaction is required in order to optimize the non-planar structures that strongly depend on hyper-conjugation effects, when methyl groups are present. To extract information on the *sp*^n^ hybridization of the carbon atoms, natural bond orbital^73,74^ (NBO) analyses were made on the SA-CASSCF optimized S_0_ and S_2_ geometries (when present). Excitation and ionization energies of the optimized S_0_ structures of benzene, toluene, and p-xylene were evaluated with coupled cluster singles and doubles (CCSD) and the aug-cc-pvdz basis set. All calculations were performed within the Gaussian09 program package.

## III. RESULTS AND ANALYSIS

### A. Computational results

**FIG. 3.**
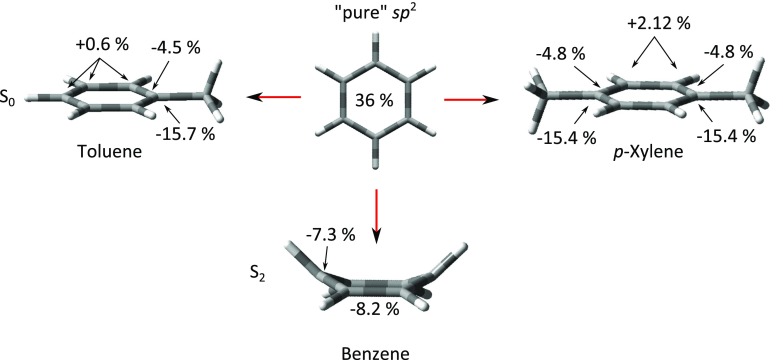
Natural bond orbital (NBO) analysis for benzene, toluene, and p-xylene. The numbers indicate the amount of s-character in the *sp*^n^-hybrid orbitals relative to the *sp*^2^-orbitals of planar benzene (corresponding to the S_0_ equilibrium structure). The changes were found to be balanced by equal changes in *p*-character. See the text for further details.

The molecular geometries that are key for the current investigation involve the Franck-Condon structures, which correspond to the S_0_ equilibrium structure, and the S_2_ equilibrium structure towards which the excited molecules will start to relax. The optimized S_0_ equilibrium structures of all three molecules were non-surprisingly found to be fully planar, while the S_2_ minimum of benzene was found to be boat-shaped with the dihedral angle of the pinnacle carbon atoms being 27.1° in agreement with the previously reported structure.^64^ The coordinates of all structures are given in the supplementary material, while the structures are shown in tube-format in Figure 3 along with the results from the NBO analysis.

NBO methods provide a means to gather intuitive chemical insight (in the framework of conceptual models from VB theory and Lewis-like structures) from highly accurate quantum chemical calculations.^74^ The current NBO analyses were used to extract information from the SA-CASSCF calculated structures in terms of the orbital-hybridization, which is a somewhat illusory property related to the mixing or symmetry-breaking of pure *s* and *p-*orbitals. Yet, it provides an intuitive and practical way of interpreting and discussing the consequences of local changes in the structures. The NBO analyses were performed with primary focus on the aromatic carbon atoms that constitute the chromophore and the carbon atoms of the methyl groups. The central results from the NBO analysis are summarized in Figure 3; the ground state optimized structures of all three molecules are shown in the top and the distorted S_2_ minimum energy structure of benzene is shown in the bottom. The numbers refer to the *s*-character of the *sp*^n^ orbitals on carbon engaging in the σ-bonds, where 36% refers to the amount *s-*character for fully planar, non-perturbed S_0_ benzene, and the ± percentages refer to the increase or decrease of *s*-character induced by methylation or geometrical distortion. Note, a decrease in *s*-character yields more *sp*^3^-like (i.e., methyl-like) carbon orbitals, which are more prone to form σ-bonds, which simultaneously implies that less *p*-character is available to form π-bonds. The amounts of *s*-character were consistently found to be balanced mainly by *p*-character, while the amounts of *d*-character were negligible.

As can be seen in Figure 3, going from a fully planar to a boat-shaped structure induced significant amounts of *s* and *p* mixing, where all carbon atoms attain sp^3^-like sp^2^-character. When the perturbation instead is invoked by methylation, the carbon atoms connected to the methyl groups attain even larger *sp*^3^-like character, while the reversed mixing is observed for the remaining carbon atoms albeit to a smaller extent. This mainly reflects that the hybrid-orbitals of a single carbon atom differ depending on whether they engage in bonds with the aromatic ring or the methyl groups and suggest that these orbitals may be more susceptible to rehybridization effects upon distortion. The extent of *s-p* mixing is found to be similar for toluene and *p*-xylene, yet with an additional carbon atom exhibiting large mixing in the case of *p*-xylene. As the S_2_ geometry optimizations of toluene and *p*-xylene did not converge, the extent of orbital scrambling when both perturbations are present could not be assessed directly; regardless, these results indicate that the methylated species are more susceptible to orbital mixing.

The lowest singlet and triplet state energies are key to understand the TRPES data. Numerous experimental and calculated energy values have been reported largely agreeing with the SA-CASSCF//CCSD/aug-cc-pvdz values calculated in the current work.^46,47,71,72^ The relevant energy values are summarized in Table I. Herein are also included ionization energies, probe photon energies as well as the expected electron kinetic energy (eKE) of the photoelectrons associated with 0–0 transitions (italicized). As apparent from Table I, the number of electronic states that can be probed in the current experiments and the associated eKE values vary between the three molecules.

**TABLE I.** Ionization (D_0_) and vertical excitation energies of benzene, toluene, and –*p*-xylene calculated using SA-CASSCF//CCSD/aug-cc-pvdz. The probe energies (*hν*_probe_) and the electron kinetic energies (eKE) of the resulting photoelectrons are also summarized. The grey/shaded regions can be white/clear. They were only included to separate the toluene results from those of benzene and p-xylene.

	Benzene		Toluene		p-Xylene	
*hν*_probe_	4.44		4.35		4.2	
D_0_	9.24		8.8		8.4	
	Vertical excitation (eV)	*eKE* (eV)	Vertical excitation (eV)	*eKE* (eV)	Vertical excitation (eV)	*eKE* (eV)
S_2_	6.03^a^	*1.2*	5.83^a^	*1.38*	5.68^a^	*1.48*
S_1_	4.72^a^	*“0”*	4.64^a^	*0.19*	4.55^a^	*0.35*
T_3_	5.6 ± 0.2^b^	*0.4 ± 0.2*	5.5 ± 0.2^b^	*1.05 ± 0.2*	5.3 ± 0.2^c^	*1.1 ± 0.2*
T_2_	4.7 ± 0.2^b^	*…*	4.5 ± 0.2^b^	*0.05 ± 0.2*	4.3 ± 0.2^c^	*0.1 ± 0.2*
T_1_	3.7 ± 0.2^d^	*…*	3.6 ± 0.2^e^	…	3.5 ± 0.2^c^	…

^a^ Reference 71.

^b^ Reference 72.

^c^ From Ref. 72 adjusted slightly to match the current calculations.

^d^ Average of Refs. 72 and 75 and in agreement with the current calculations.

^e^ Average of Refs. 72 and 76 and in agreement with the current calculations.

### B. Experimental results

**FIG. 4.**
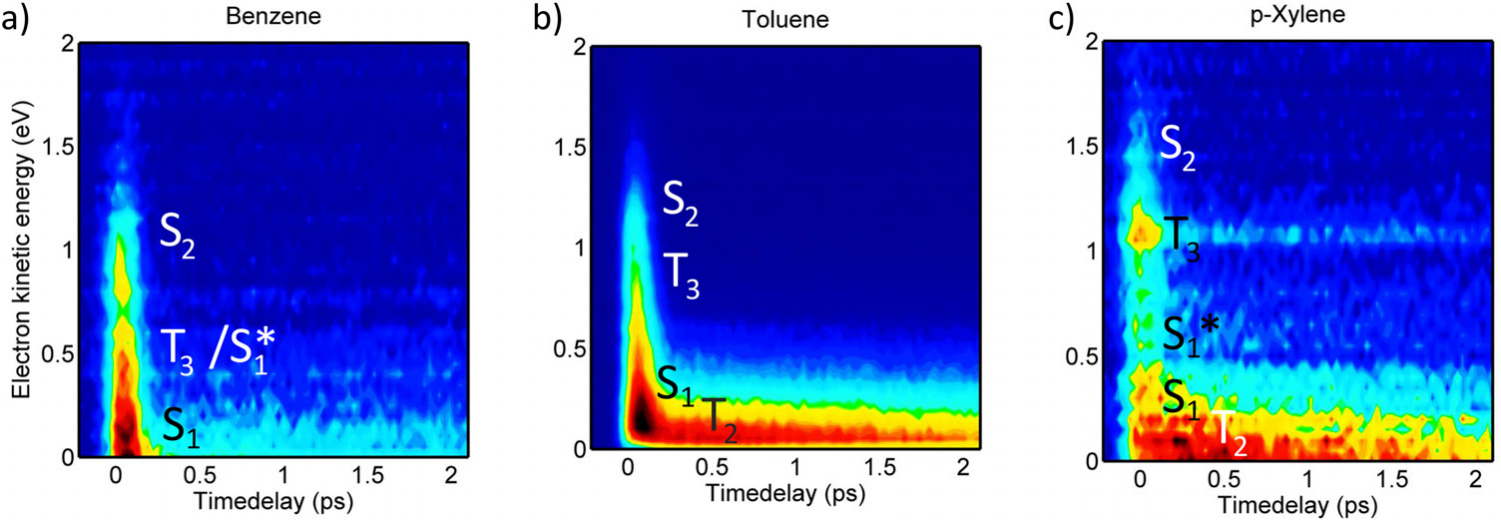
Contour plots showing the TRPES data recorded for (a) benzene (200 nm + 279 nm), (b) toluene (200 nm + 286 nm), and (c) p-xylene (200 nm + 295 nm).

The photoinduced S_2_ dynamics of benzene, toluene, and *p*-xylene were studied in 1 pump + 1 probe photon photoexcitation and ionization schemes, and the probe energies and intensities were chosen to minimize parallel multiphoton processes. Background subtraction confirmed that the contribution from parallel processes is negligible. Representative contour plots associated with the TRPES experiments on benzene, toluene, and p-xylene are shown in Figures 4(a)–4(c); eKEs are shown on the y-axis while the x-axis indicates the temporal delay between the laser pulses. The TRPES data show significant similarities for the three molecules: (i) all contour plots exhibit large diffuse energy features near time-zero consistent with previously reported photoelectron spectra of these molecules,^1,3,4,66^ (ii) rapidly decaying signal intensities are observed in large energy areas, and (iii) to long time-delays signal intensity is primarily observed at low eKEs. The contour plots also exhibit clear differences: (i) p-xylene exhibits a clear signal at 0.95–1.2 eV not immediately visible for benzene and toluene, (ii) progressively (but only slightly) later arrival times and slower dynamics are observed in the low eKE regimes upon increasing methylation (this is most prominent near zero eKE of toluene and p-Xylene), and (iii) more distinct spectral features at early times for benzene and p-xylene as compared to toluene, i.e., the diffuse broad spectra near time-zero show more structure in the data sets of benzene and p-xylene.

By comparison with Table I, S_2_ features are expected in the high eKE range of the spectra, and possible T_3_ features are expected at intermediate energies just below S_2_. Only the highly vibrationally excited edge of S_1_ can be probed for benzene and should appear near zero eKE values, while S_1_ is expected 0.6–0.7 eV below T_3_ in toluene and *p*-xylene. For benzene and *p*-xylene, T_2_ should appear near eKE = 0–0.2 eV. The broad and diffuse spectral features observed partly result from a wide envelope of vibrational ionizations^1,3,4,66^ but possibly also due to electronic transitions occurring faster than the temporal resolution of the laser (≈150 fs) yielding broadened and diffuse features.

The temporal evolution of the photoelectron spectra was evaluated by fitting the channel integrated and normalized signals. Due to the continuous spectral features, it is not immediately apparent how to integrate the spectra, and thus several different integration regimes were explored to optimize a fair treatment of the data. The final integrations were based on the observed dynamics and further chosen to match the energy areas expected for the respective singlet and triplet states given in Table I. In each case, energy regions representing, respectively, S_2_, S_1_^*^/T_3_ (where * denotes vibrational excitation), and S_1_ were evaluated. Additionally, energy regimes corresponding to T_2_ were evaluated for toluene and p-xylene, and for p-xylene a highly excited S_1_* area was also explored as signal intensity was observed in an area mainly matching highly excited S_1_ (0.5–0.95 eV). The integrated transients were fit to a sum of exponential functions convoluted with a Gaussian response function.

In the cases where relaxed S_1_ could be probed (toluene and p-xylene), the S_1_ transients exhibit long lifetime components, which here is taken into account by adding a constant offset after time-zero as the signal intensity extends far beyond (100s of nano-second) the picosecond time regime explored here.

**FIG. 5.**
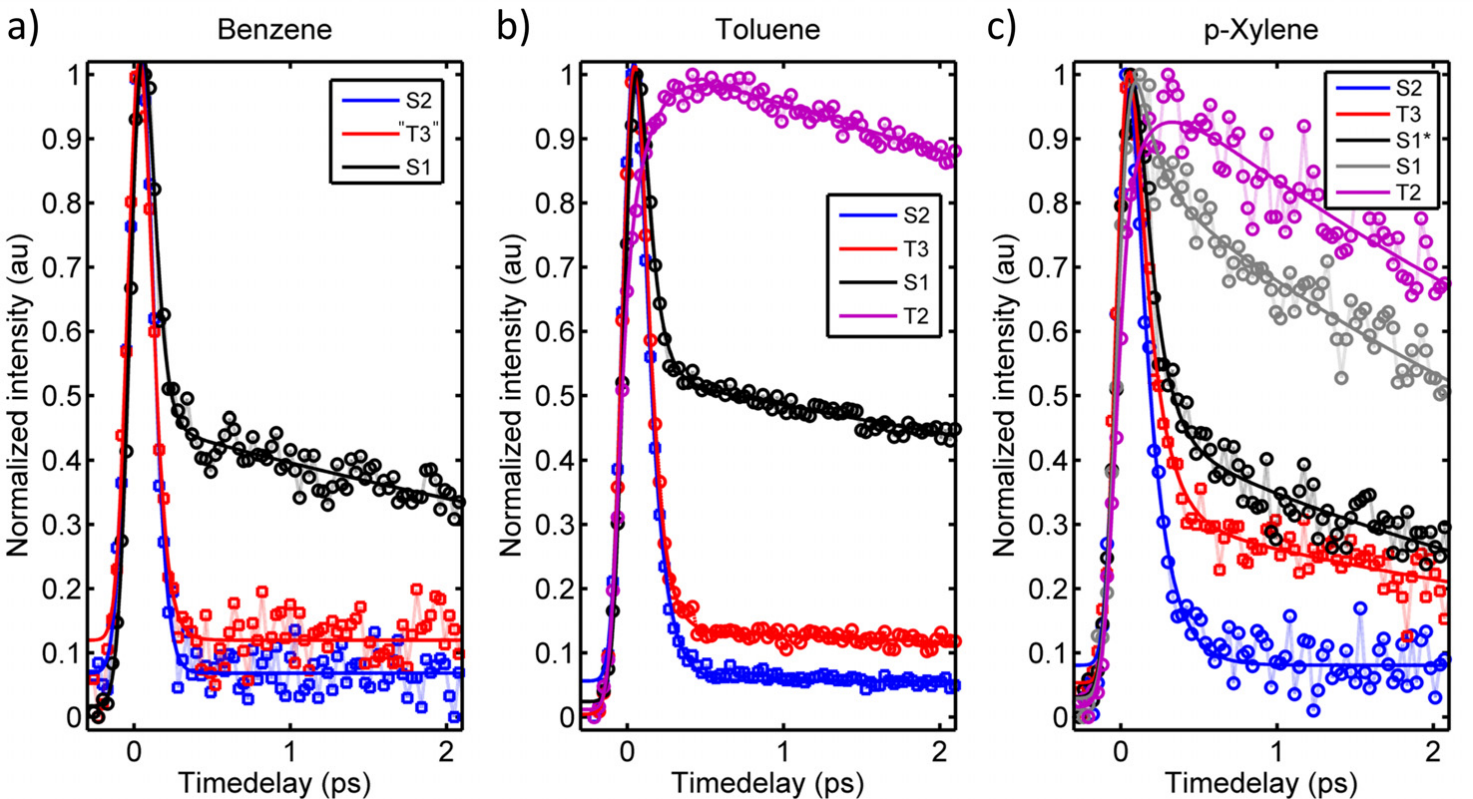
Integrated and normalized transients for (a) benzene, (b) toluene, and (c) p-xylene including their associated fits. The fitted time components are summarized in Table II. Legends indicate tentative assignments based on the eKE ranges and the calculated energies, the validity of the assignments are discussed in the text.

The number of exponential functions required to fit the data varied across the spectrum; the fitted time-constants along with the integrated energy regions are summarized in Table II, where τ_rise_ indicates rise-times, τ_fd_ and τ_sd_ denote fast and slow decays, respectively, while “- -” indicates that no extra exponential function was needed, i.e., cross correlation limited rise-times or mono-exponential decays. Attempts to fit the (ultrafast) rising behavior observed in the spectral regions of T_3_ and S_1_ yielded rise times 5–15 fs in all cases, indicating that the instrumental time-resolution (150 fs) is insufficient to capture the rise of these spectral features. The rise-times are therefore denoted with “- -” in Table II. It should be mentioned that the uncertainties indicated are solely based on the fitted values and included to reflect the variation in data quality. All time-constants shorter than the cross-correlation of ≈150 fs can only be concluded to be faster than or equal to approximately 150 fs, though a reasonable agreement with previous results is observed even for the shortest time-components (in the cases where previous estimates exist).^65,66^ The transients including the associated fits are shown in Figures 5(a)–5(c). The assignments denoted in Table II and Figures 5(a)–5(c) are tentative and based on the calculated and previously reported energy values (Table I). While the spectral manifestation of, for example, the T_3_ state of benzene is much more ambiguous than the equivalent of, e.g., *p*-xylene, integration of the T_3_ region of benzene is necessary in order to allow for comparative discussion. For the ease of the discussion, the signals will therefore be referred to accordingly in the remainder, and the validity and rationality of the assignments are discussed in Section IV.

**TABLE II.** Integrated energy regions and the associated rise (τ_rise_) and decay (τ_fd_ or τ_sd_) times of the TRPES data on benzene, toluene, and p-xylene, “- -” denotes that no extra exponential function was needed to fit the data. Tentative assignments are indicated and discussed in the text, e.g., in the case of benzene the assignment of T_3_ is non-conclusive and the signal intensity should likely be ascribed excited S_1_^**^ instead. The grey/shaded regions can be white/clear. They were only included to separate the toluene results from those of benzene and p-xylene.

Benzene				Toluene				p-Xylene			
eKE (eV) (state)	τ_rise_	τ_fd_ (fs)	τ_sd_ (ps)	eKE (eV) (state)	τ_rise_ (fs)	τ_fd_ (fs)	τ_sd_ (ps)	eKE (eV) (state)	τ_rise_ (fs)	τ_fd_(fs)	τ_sd_ (ps)
0.75–1.7 (*S_2_*)	- -	40 ± 10	_- -_	0.8–1.75 (*S_2_*)	- -	75 ± 5	- -	1.25–2 (*S_2_*)	- -	100 ± 20	- -
0.5–0.7 (S_1_^**^ or T_3_)	- -	55 ± 10	_- -_	0.6–0.8 (S_1_^*^ or T_3_)	- -	75 ± 5	14 ± 5	0.95–1.2 (*T_3_*)	- -	180 ± 20	3.9 ± 0.8
0–0.5 (S_1_^*^)	- -	50 ± 10	6.3 ± 0.7					0.5–0.95 (S_1_^*^)	- -	110 ± 20	3.3 ± 0.4
				0.1–0.6 (*S_1_*)	- -	75 ± 5	10 ± 0.5	0.15–0.45 (*S_1_*)	- -	3200 ± 200	Off set
				0–0.1 (*S_1_ or T_2_*)	150 ± 15	12 ± 0.6	Off set	0–0.15 (*T_2_*)	200 ± 50	5000 ± 400	*- -*

## IV. DISCUSSION

**FIG. 6.**
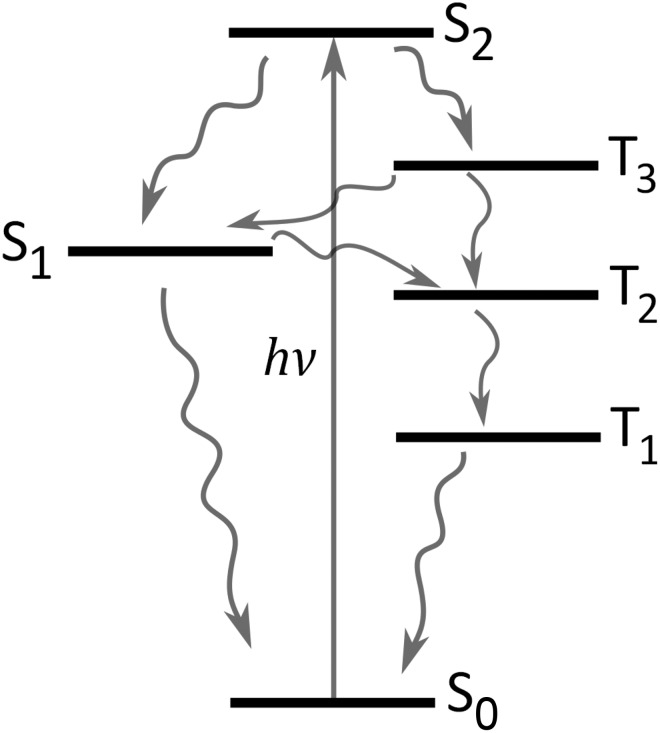
General and simplified Jablonski diagram showing what pathways are available from the Franck-Condon geometry of S_2_ based on energetic considerations.

The general similarity between the contour plots of the TRPES data of benzene, toluene, and p-xylene suggests that their S_2_ excited state deactivation dynamics are similar. Yet, the three contour plots are more different than what immediately would be expected. The most striking difference is the presence of a distinct signal at 0.95–1.2 eV for *p*-xylene, which is not clearly apparent for benzene and toluene. The observed differences are likely due to subtle changes invoked by methylation that affect the relative prominence of the available S_2_ deactivation channels for each of the three molecules. A simplified general Jablonski diagram representative for all three molecules is shown in Figure 6, which only includes the most obvious pathways available, while more complex pathways are omitted, as Figure 6 appears sufficient to discuss the current data. The deactivation pathways drawn are energetically available for all three molecules, but the differences in the contour plots suggest that the channels are not equally active. While we acknowledge that the observed differences could be due to varying efficiencies of projection of the dynamics on to the observable (for example, different ionization cross sections), we will in the following discuss which and why certain channels could be more operative in some molecules relative to the other. Subsequently, the generalized Jablonski diagram of Figure 6 is resumed for each of the three species.

### A. S_2_ deactivation: Competing IC and ISC

**FIG. 7.**
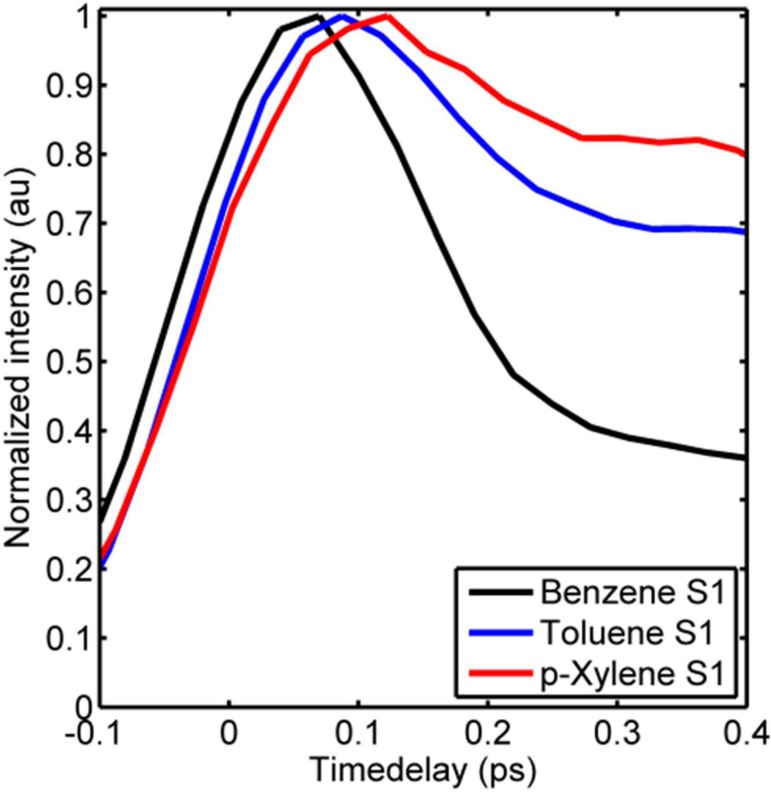
S_1_ transients for benzene (black), toluene (blue), and p-xylene (red) to early times. The zoom in highlights the difference appearance times of the three transients.

Based on energetic proximity arguments and previous investigations,^41,42,46,47,66^ T_3_ and S_1_ are the most likely receiver states for S_2_ deactivation. The most obvious and conventionally expected S_2_ → S_1_ relaxation pathway is posited to be a prefulvenic half-boat mode^47,66^ and IC is reported to proceed within 40–60 fs.^46,47,65,66^ This is consistent with the current observations of cross-correlation limited decays that can be fitted to 40–100 fs time components (Table II); the slightly longer timescale for *p*-xylene may be due to slight spectral overlap with the T_3_ signal. The rapid cross-correlation limited decay of S_2_ concomitant with the observation of signal intensity in the S_1_ energy regimes that appear within the cross-correlation of the pump and probe pulses agrees well with a rapid S_2_ → S_1_ IC process as previously proposed.^46,47,65,66^ Close inspection of the early time dynamics in the S_1_ regions shows that the S_1_ signals reach their maximum intensities at slightly different times for the different molecules; Figure 7 shows a zoom in on the S_1_ transients to early times, and as can be seen, the appearance time of the S_1_ signal increases in the order (from early to late) benzene < toluene < p-xylene. It should be mentioned that only the upper edge of the S_1_ state of benzene could be probed, and thus this observation should be interpreted cautiously. Nonetheless, the trend is consistent with the results from, e.g., Suzuki *et al.* on benzene and toluene^66^ and is interpreted to reflect the higher frequency of the prefulvenic mode for benzene than the methylated analogues yielding a faster S_2_ → S_1_ IC for benzene. The observation of a ca. 3–10 ps decay of the energy region corresponding to vibrationally excited S_1_ is also consistent with earlier work, where it was ascribed IC to S_0_.^65^ Vibrationally excited S_1_ may undergo intramolecular vibrational energy redistribution concurrently with IC to S_0_ obscuring the decay times somewhat. Since the IC channel has been the focus of previous studies, we will turn focus towards the possibility and manifestation of S_2_ → T_3_ transitions in the following.

The observation of an S_2_ → S_1_ transition faster than 150 fs implies a highly non-statistical process mainly activating a few reaction coordinates largely determined by Franck-Condon factors and the topology of the potential energy surface near the Franck-Condon region.^44^ The current and previous^64^ calculations predict the S_2_ equilibrium structure to be boat-shaped and distorted out-of-plane relative to the Franck-Condon geometry (Figure 3), and thus early activation of prefulvene-like or boat-shaped modes is expected.^66^ The highly non-statistical nature of the S_2_ → S_1_ IC process suggests that any competing ISC process to form T_3_ could occur along the same vibrational coordinate. This is similar to what has been proposed for the IC vs. ISC competition for S_1_ deactivation of benzene (namely, that the transition to S_0_ and T_2_ occurs along similar coordinates^1,3,4^) and further corroborated by theoretical studies by Cogan *et al.* showing a tendency for the spin-orbit coupling to increase at triple crossing points,^77^ e.g., where a crossing between two singlet surfaces coincides with a crossing to the triplet manifold. Importantly, the prefulvene-like modes break the symmetry of the aromatic ring and enable mixing of σ-character into the π-system.^11^ The NBO analysis summarized in Figure 3 shows that upon distortion to the boat-shaped S_2_ minimum of benzene, the carbon orbitals undergo significant rehybridization in that all carbon atoms attain increased *sp*^3^–*sp*^2^-like characters, implying that the π-bonds become more σ-like and vice versa. Distortion and associated rehybridization thereby open the possibility for a partly allowed ISC process involving both σ and π-orbitals. For toluene and *p*-xylene, the methyl carrying carbon atoms already possess notable amounts of sp^3^-like character in the planar geometries indicating that these carbon-atoms may be even more susceptible to σ and π mixing.

Considering the observation of ultrafast S_2_ depletion, potential formation of T_3_ should also likely occur within the cross-correlation of the two laser pulses. The distinct signal observed between 0.95 and 1.2 eV for *p*-xylene matches the expected energy region for T_3_ and is observed to appear within the cross correlation similar to the S_1_ signal. The observed signal between 0.95 and 1.2 eV does not match other electronic states of *p*-xylene energetically or dynamically (though the upper edge may overlap slightly with S_2_). We therefore interpret this feature as an unusually clear manifestation of an upper triplet state of *p*-xylene. As recently discussed,^43^ rapid IC of upper triplet states often follow immediately after ISC due to high densities of states in the triplet manifold which in combination with (often) low triplet quantum yields make upper triplet states challenging to observe experimentally. The T_3_ signal observed for *p*-xylene is thus surprisingly clear. Turning to benzene and toluene similar evidence for T_3_ states is less prominent. In both cases, the spectra show signal intensity in the energy regions of the respective triplet states, but the features are much less distinct. This might be due to several effects involving smaller triplet yields and/or more rapid decay of T_3_ as further discussed in Section IV B. At this point, S_2_ can tentatively be concluded to decay to both S_1_ and T_3_ for p-xylene at notable (yet, not quantifiable) amounts, and only the S_2_ → S_1_ channel is clear for toluene and benzene though some portions of the S_2_ populations may convert to the triplet manifold; further indications of triplet formation or lack of the same are discussed in Section IV B.

### B. T_3_ formation and deactivation efficiencies affecting the T_3_ manifestation

The spectral manifestation of T_3_ depends on several effects involving quantum yields, photoionization cross-sections, lifetimes, and potentially overlapping features obscuring the signal. Reliable quantum yields are hard to estimate as photoionization cross-sections are not easily predicted. Instead the discussion will first focus on the T_3_ activation mechanisms thereby invoking the lifetimes and potential overlapping spectral features. The T_3_ state of p-xylene is observed to decay bi-exponentially with time constants of ca. 180 ± 20 fs and a small amplitude decay component of 3.9 ± 0.8 ps. The spectral regions corresponding to T_3_ of benzene and toluene in both cases decay cross-correlation limited. The possible deactivation pathways of T_3_ involve IC in the triplet manifold and back ISC to the singlet manifold (Figure 6). There is no immediate evidence of the latter process; S_1_ is observed to rise within the cross-correlation for all molecules with no additional slower components. We therefore turn focus to IC in the triplet manifold instead.

**FIG. 8.**
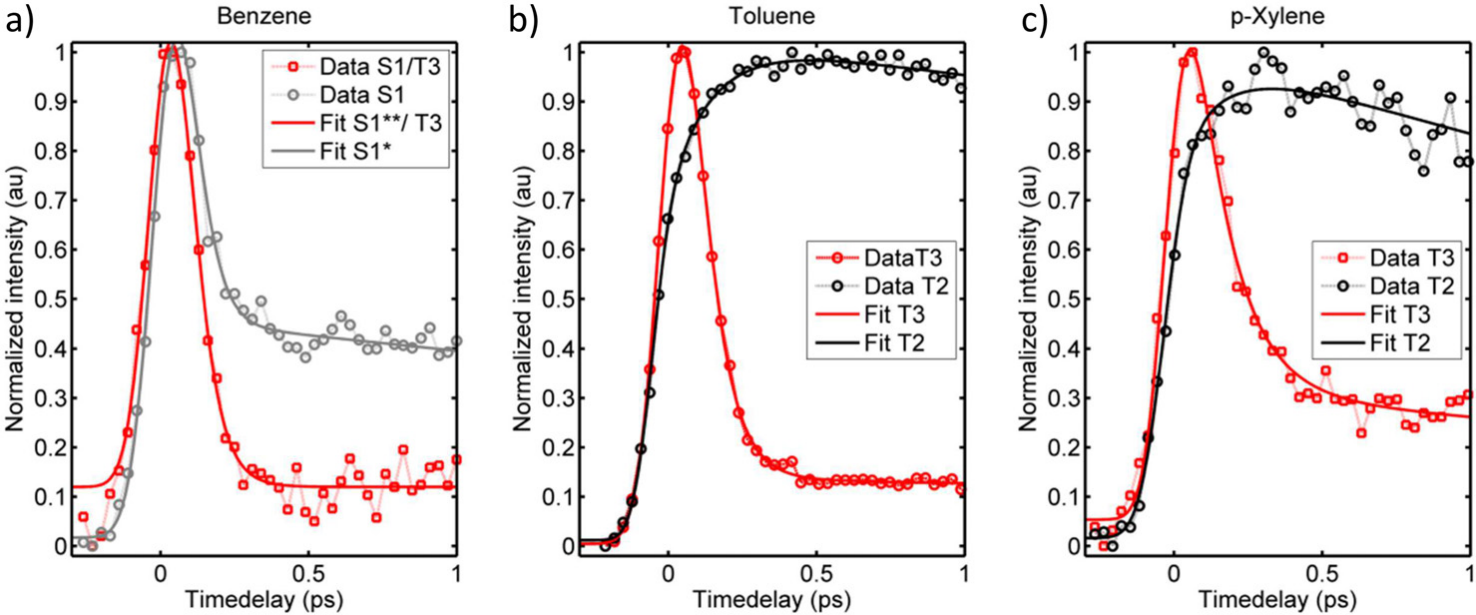
Fitted transients of the energy regions corresponding to the T_3_ state (red) and the 0–0.15 eV regions (grey/black) of (a) benzene, (b) toluene, and (c) p-xylene. For toluene and p-Xylene, the low energy regions match the T_2_ energies, and the red and black transients exhibit mirroring dynamics. Similar dynamics is not observed for benzene (a).

Signals corresponding to ionization out of T_2_ should appear in the lowest eKE region of the spectra of p-xylene and toluene (Table I), while the probe photons are not sufficiently energetic to probe the T_2_ state of benzene. Interestingly, rising features are observed for both toluene and p-xylene in the low eKE (≤0.15 eV) energy regimes. For p-xylene, the rise-time in this energy region can be fit to 200 ± 50 fs thereby mirroring the fast decay of T_3_ (180 ± 20 fs), while the corresponding rise-time for toluene can be fit to 150 ± 15 fs, consistent with the cross-correlation limited decay of T_3_. The matching rise and decay components indicate that these low eKE features can be ascribed T_2_. We note that for toluene portions of the T_2_ spectrum may overlap with the spectrum of S_1_; however, the rise of the transient is notably slower than the expected (and observed) cross-correlation appearance of the S_1_ signal. The rising spectral features are visible in both the contour plots (Figure 4) and on the integrated transients (Figure 5). The transient complementarities are more clearly visible in Figures 8(b) and 8(c) where only the T_3_ and T_2_ transients are shown for toluene and p-xylene, respectively. Figure 8(a) shows the transients of the T_3_ region and the 0–0.15 eV regions for benzene to highlight the absence of similar dynamics in the low energy part of the spectrum for benzene (i.e., it appears unlikely that the rising features are due to parallel processes induced by the two laser beams). At this point, we therefore ascribe the approximately 200 fs (for *p*-xylene) and 150 fs (for toluene) rise and decay components to the T_3_ → T_2_ transition.

The tendency of the T_3_ → T_2_ IC rate in the order toluene > p-xylene matches previous TRMS experiments on *o-*, *m-, p*-xylene and toluene assessing the S_3_ → S_2_ IC.^46,47^ The relative S_3_ → S_2_ IC rates were found to be toluene > *o*-xylene ≈ *m*-xylene > *p*-xylene, which (corroborated by knowledge on the equilibrium structures) was taken as an indication that the full-boat out-of-plane distortion mode facilitated the IC process. If the same mechanism transfers to the triplet manifold (considering the equivalent electronic characters of the S_3_ ↔ T_3_ and S_2_ ↔ T_2_ states, it appears reasonable to compare the rates and mechanisms in the respective multiplicity manifolds), the current observation of relative T_3_ → T_2_ IC rates in the order toluene < *p*-xylene is consistent with the expected. Such an IC mechanism should put benzene in front of toluene as the fastest decaying T_3_ state, provided that the T_3_ state of benzene is formed. Rapidly decaying T_3_ should thus only be observed in the time-zero region.

**FIG. 9.**
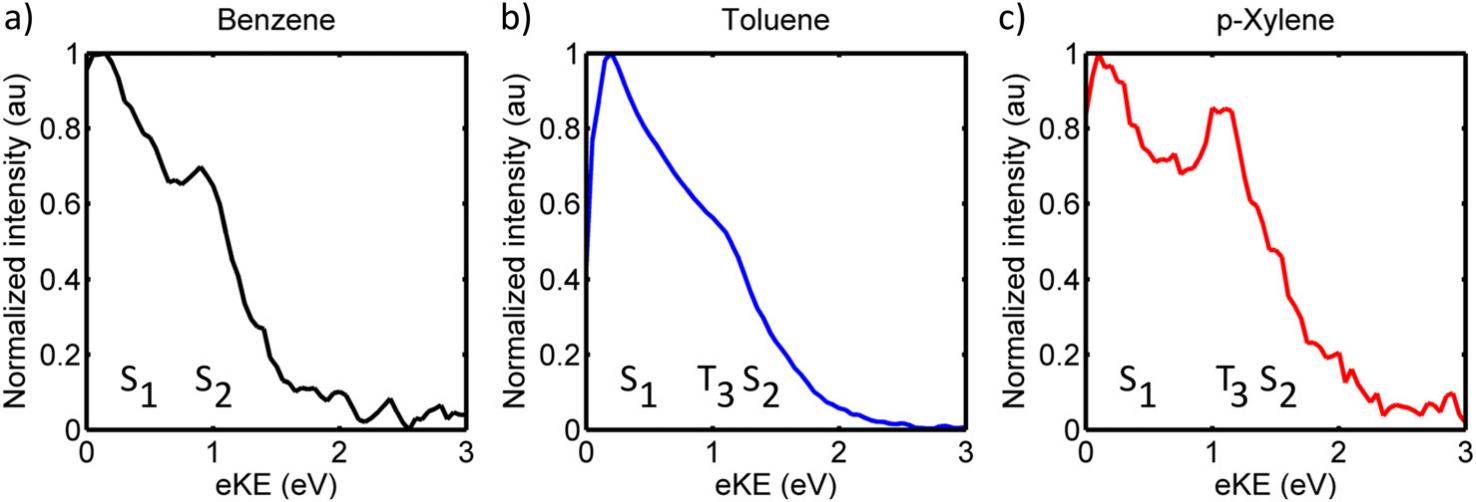
Time-zero slices of the TRPES contour plots (shown in Figure 4) of (a) benzene, (b), toluene, and (c) p-xylene.

In this respect, it is appropriate to inspect the time-zero spectra more thoroughly. As mentioned in Section III B, the time-zero spectra differ; this is apparent in the contour plots (Figure 4) but more clearly at the time-zero slices shown in Figures 9(a)–9(c). Benzene and p-xylene show more distinct features than toluene, which exhibits one broad diffuse band with very little structure. This could be an indirect indication that the T_3_ yields increase in the order from benzene < toluene ≤ *p*-xylene (as further discussed in Section IV C). Assuming this trend is correct the clear features in benzene (corresponding to S_2_ and S_1_) result from minimal S_2_–T_3_–S_1_ overlap due to very little (if any) T_3_ formation. The T_3_ yield of toluene is possibly higher compared to that of benzene, and toluene therefore suffers more from spectral congestion. *p*-Xylene also undergoes ISC to T_3_, and the T_3_ state furthermore lives longer than the T_3_ state of toluene, and *p-*xylene therefore shows an actual discernible spectral feature along with the (slightly overlapping) broad S_2_ and S_1_ features.

### C. Unifying picture and the effect of methylation

**FIG. 10.**
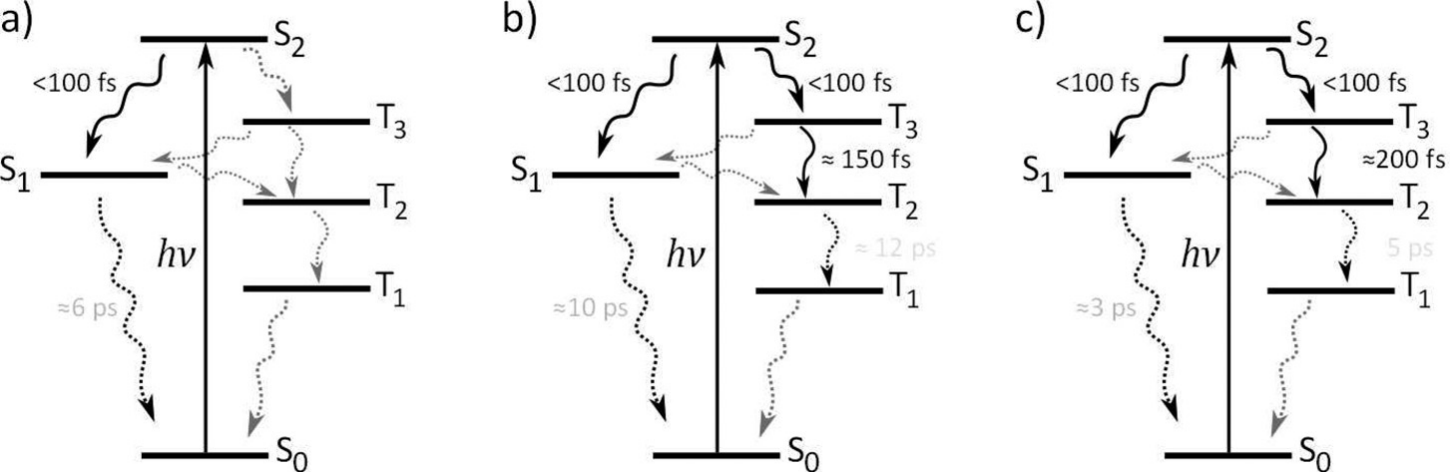
Unifying interpretation of the S_2_ decay dynamics of (a) benzene, (b) toluene, and (c) p-xylene. The black arrows indicate the transitions observed in the data (precursor and successor), the dotted black lines indicate processes that only are indirectly observed as decaying transients of the precursor, and the grey dotted lines indicate inactive processes.

Collecting the observations on the individual TRPES data sets and the differences between them, the combined interpretation can be summarized as illustrated in Figures 10(a)–10(c). The interpretation presented in Figure 10(a) pertaining to the data for benzene illustrates that S_2_ primarily deactivates via ultrafast IC to S_1_. The lack of clear T_3_ signal (as compared to *p*-xylene) and the more distinct time-zero spectrum (as compared to toluene) suggests that negligible amounts of T_3_ are formed for benzene. Figure 10(b) shows that toluene deactivates S_2_ via ultrafast IC to S_1_ and likely also via ultrafast ISC to T_3_ with lifetimes shorter than 150 fs. The contribution of the triplet manifold is manifested by the ultrafast decaying signal in the T_3_ energy regime yielding a diffuse feature overlapping with S_2_ and S_1_ on the time-zero spectrum, and by the observation of a rising component of about 150 fs in the T_2_ energy region. The T_2_ signal does not fully match other electronic states of toluene in eKE or in appearance time. This assignment is corroborated by the clearer triplet signals of *p*-xylene: Figure 10(c) shows that the S_2_ state of *p*-xylene undergoes ultrafast IC and ISC similar to that of toluene. The T_3_ signal of p-xylene is more distinct than that of toluene due to the longer T_3_ lifetime. Whether the strong T_3_ signal also is due to a higher triplet yield of *p*-xylene compared to toluene can however not be concluded from the present data. T_3_ undergoes bi-exponential decay with the majority converting to T_2_ on a ≈200 fs time-scale which is manifested in the data by matching rise and decay components. Whether the remaining part of T_3_ also undergoes IC to T_2_ is unclear as a potential bi-exponential T_2_ rise would be obscured by simultaneous T_2_ decay. The observation of slightly faster T_3_ → T_2_ IC for toluene (≈150 fs) compared to p-xylene (≈200 fs) is consistent with the activation of a full-boat motion as previously suggested for the electronically equivalent S_3_ → S_2_ transition.^46^

From this interpretation, methylation is found to increase the ISC yield (possibly but not necessarily progressively upon further methylation). This interpretation agrees well with the results from the NBO analysis based on the degree of *s* and *p*-mixing (Figure 3). In the framework of the VB theory, the overlap between two orbitals is expected to be better if the orbitals are alike, and orbital hybridizations are expected to adjust accordingly. This is consistent with the current NBO-analysis, where the *sp*^n^-orbitals of the carbon atoms which connect the ring with the methyl groups of toluene and *p-*xylene are found to be more sp^3^-like compared to the remaining *sp*^2^ carbon orbitals of the aromatic system and compared to unsubstituted benzene (Figure 3). This indicates that the carbon atoms linking the aromatic and alkyl-moieties are more susceptible to hybridization-effects when the molecules distort on the excited state surface.

As mentioned in the introduction and investigated in, for example, Ref. 11, the probability of ISC depends on mixing of and σ and π orbitals when no obvious El-Sayed type transitions are present. For aromatic hydrocarbons this is possible along out-of-plane modes,^11,78^ and as indicated here in both the experimental and theoretical results the effect increases when methyl-substituents are present. Thus, the role of the methyl group is both to guide the axis of the out-of-plane distortion (preferentially along the axis containing methyl groups, due to the stabilization effect of the methyl-groups on the pseudo-radicaloid prefulvenic structure, Figure 2) and to increase the σ-character mixing into the bonds of the pinnacle carbon atoms thereby enhancing the ISC probability. This is in full agreement with the stronger spectroscopic evidence for triplet formation in toluene and p-xylene as compared to benzene.

## V. CONCLUSION

Time-resolved photoelectron spectroscopy (TRPES) has been used to probe the competition between intersystem crossing (ISC) and internal conversion in benzene, toluene, and p-xylene upon excitation to S_2_. All molecules were found to exhibit ultrafast S_2_ decays. For benzene, the excited state population appears to mainly convert internally in the singlet manifold as deduced from the presence of S_1_ signal and concomitant absence of clear triplet signal in the TRPES data. It is possible that the triplet yield was too low to be apparent in the data in the case of benzene. This contrasts the case of the methylated benzene derivatives, where ISC to the triplet manifold is indicated by the spectral observations of T_3_ and T_2_ signals. For *p*-xylene, the prominence of the triplet signal was unusually clear. In both cases, T_3_ was observed to appear within the cross-correlation of the experiment (<150 fs), which is consistent with the cross-correlation limited decay of S_2._ The T_3_ signals decays of <150 fs (toluene) and ≈200 fs (*p*-xylene) were observed to mirror the rising components of the T_2_ signals as clear indicators of population transfer. The key vibrations mediating both IC in the singlet and triplet manifolds as well as the ISC pathway involve prefulvene-like out-of-plane distortions.

The TRPES investigations were corroborated by quantum chemical calculations. Natural bond orbital analysis of the SA-CASSCF optimized structures indicated that the carbon atoms distorting out of the aromatic plane during the transition undergo significant amounts of rehybridization along the reaction coordinate. The extent of orbital mixing was found to increase upon methylation, with the most significant rehybridization effects being localized on the methyl-carrying carbon atoms. Methyl-substituents are therefore proposed to both enhance the ISC probability and to direct the excited state dynamics to involve the molecular axis containing the methyl-groups in the case of toluene and *p*-xylene. The consistent results from the TRPES and theoretical investigations imply that ISC can occur even in system where no obvious ISC mechanism appears to be available in the Franck-Condon area.

## VI. SUPPLEMENTARY MATERIAL

See supplementary material for the coordinates of the optimized structures of benzene, toluene, and *p-*xylene.
